# Ewing sarcoma of the adrenal gland: a case report and review of the literature

**DOI:** 10.1186/s13256-018-1601-7

**Published:** 2018-03-16

**Authors:** Hanane Eddaoualline, Khadija Mazouz, Bouchra Rafiq, Ghizlane EL Mghari Tabib, Nawal EL Ansari, Rhizlane Belbaraka, Abdelhamid El Omrani, Mouna Khouchani

**Affiliations:** 1 Radiation Oncology Department, Mohammed VI University Hospital, Marrakech, Morocco; 2 Endocrinology Department, Mohammed VI University Hospital, Marrakech, Morocco; 3 Medical Oncology Department, Mohammed VI University Hospital, Marrakech, Morocco

**Keywords:** Ewing sarcoma, Peripheral primitive neuroectodermal tumor, Adrenal gland neoplasms

## Abstract

**Background:**

Ewing sarcoma/primitive neuroectodermal tumor is a family of highly malignant proliferation of neuroectodermal origin, most often skeletal, adrenal localization is extremely rare. Only few cases have been reported in the literature. Classical management includes radical surgery with adjuvant chemotherapy or radiotherapy or both. This case report is the only one where recurrence was surgically removed, and it confirms the importance of adjuvant treatment, and the efficacy of neoadjuvant chemotherapy.

**Case presentation:**

We report the case of a 23-year-old Moroccan woman presenting with flank pain. An abdominal computed tomography scan showed a large and enhancing left suprarenal mass. After radical nephrectomy, histologic examination revealed a small round cell proliferation. The diagnosis of Ewing sarcoma was confirmed by molecular analysis; time to final diagnosis was 5 months due to financial and coordination issues. Computed tomography (on an asymptomatic patient) revealed a locoregional recurrence, our patient received 12 cycles of the vincristine, doxorubicin and cyclophosphamide/ifosfamide and etoposide protocol used in an alternating schedule, with partial radiologic response (62%) and pathologic complete response, then underwent adjuvant radiotherapy of 45 Gy. The young women is still in remission after 36 months of follow-up.

**Conclusions:**

Our patient had an early recurrence due to absence of adjuvant treatment, but did respond well to neoadjuvant chemotherapy with a pathologic complete response. Management of adrenal Ewing sarcoma could be extrapolated from skeletal one with good outcomes even in locoregional recurrence.

## Background

Ewing sarcoma/primitive neuroectodermal tumors (ES/PNET) was first described in 1921 as a family of tumors characterized by small round cell proliferation and characteristic translocation t(11;22). It is the second most common bone tumor in children and adolescents. The prevalence of extraskeletal ES ranges between 15 and 20%; primitive adrenal ES tumor is very rare. To date, there have been only 28 cases reported in the literature; here we report another case with a review of the literature.

Most of the cases reported in the literature were managed with initial surgery followed by adjuvant chemotherapy or radiotherapy or both. Here, we present the case of a young woman with classical presentation of adrenal ES, but who did not receive adjuvant therapy after radical nephrectomy. Evolution confirmed the importance of adjuvant treatment, but also the efficacy of the vincristine, doxorubicin and cyclophosphamide/ifosfamide and etoposide (VAC/IE) protocol that allowed a pathologic complete response. This case report is the only one where recurrence was surgically removed.

## Case presentation

Our patient is a young Arab needlewoman of 23 years old, unmarried, of middle socioeconomic status, nonalcoholic, a nonsmoker without relevant past medical history, no neoplasm was reported in the family.

Her symptoms included progressive abdominal pain and left flank distension with a conserved general status and regular menstrual cycle, without exophthalmia or any sign of hypertension or flush syndrome. Our patient had lost 1 kg in 1 month, she was taking nonsteroidal anti-inflammatory drugs (NSAIDS) with moderate pain improvement and consulted 26 days after her first symptom.

Clinical examination found a performance status (PS) 1 patient, weighing 60 kg and 158 cm tall, her temperature at admission was 37.3°, with normal blood pressure values (125/80 mmHg) and cardiac frequency (87 beats/min), without neurological abnormality or any sign of Cushing syndrome.

Computed tomography (CT) scans revealed an invasive suprarenal mass of 14 × 11 cm moderately enhanced after contrast administration, with regional adenomegaly, without venous thrombosis or distant abnormality.

Endocrinal analysis was negative: eliminating first a pheochromocytoma (normetanephrine blood level: 1.55 μmol/24 h, metanephrine: 0.32 μmol/24 h), and a corticosurrenaloma (urinary free cortisol at 370 μg/L, and two negative dexamethasone suppression tests). Cervical ultrasound found thyroid nodules of the Thyroid Imaging Reporting and Data System (TIRADS) 4A classification, with negative fine-needle-aspiration cytology, and normal thyroid function analysis.

A complete blood count showed a hypochromic anemia of 11 g/dL, with normal hepatic and renal function.

The young woman underwent an open radical nephrectomy.

**Fig. 1:**
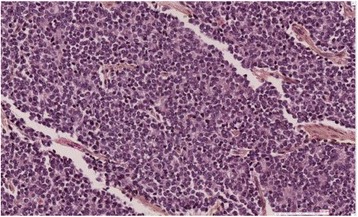
Malignant small cell proliferation in hematoxylin-eosin-saffron staining, magnification ×20

**Fig. 2:**
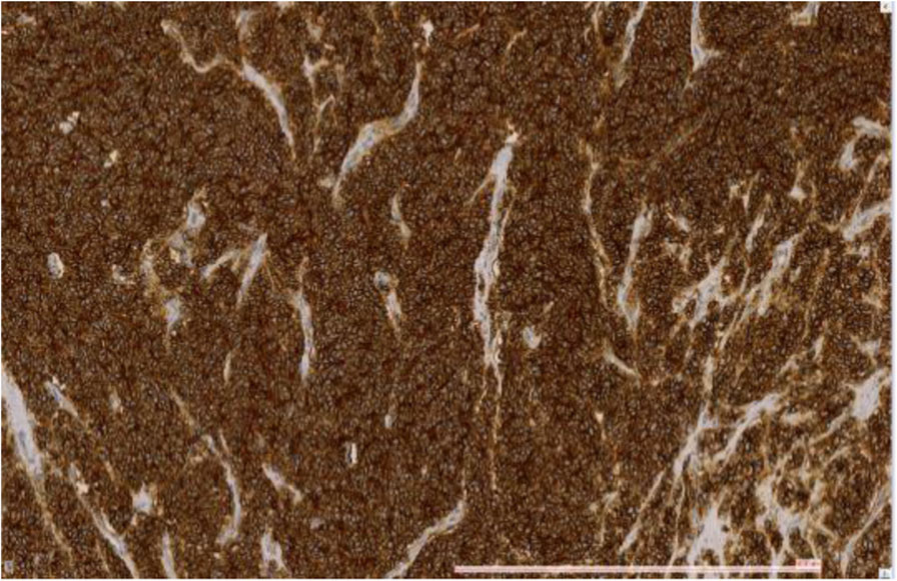
Diffuse CD99 positive staining

Pathological analysis showed a well-limited, whitish mass of 14 × 10 cm with a central fibrous zone, replacing the entire adrenal gland and infiltrating the superior renal pole and pararenal fat with negative margins, consisting of undifferentiated monomorphic small cells (Fig. 1). One lymph node among the 13 removed was metastatic, an immunochemical profile revealed an intense expression of CD99 (Fig. 2) and vimentin, and focal expression of synaptophysin and CD56, Ki67 Antigen expression was 70%.

**Fig. 3:**
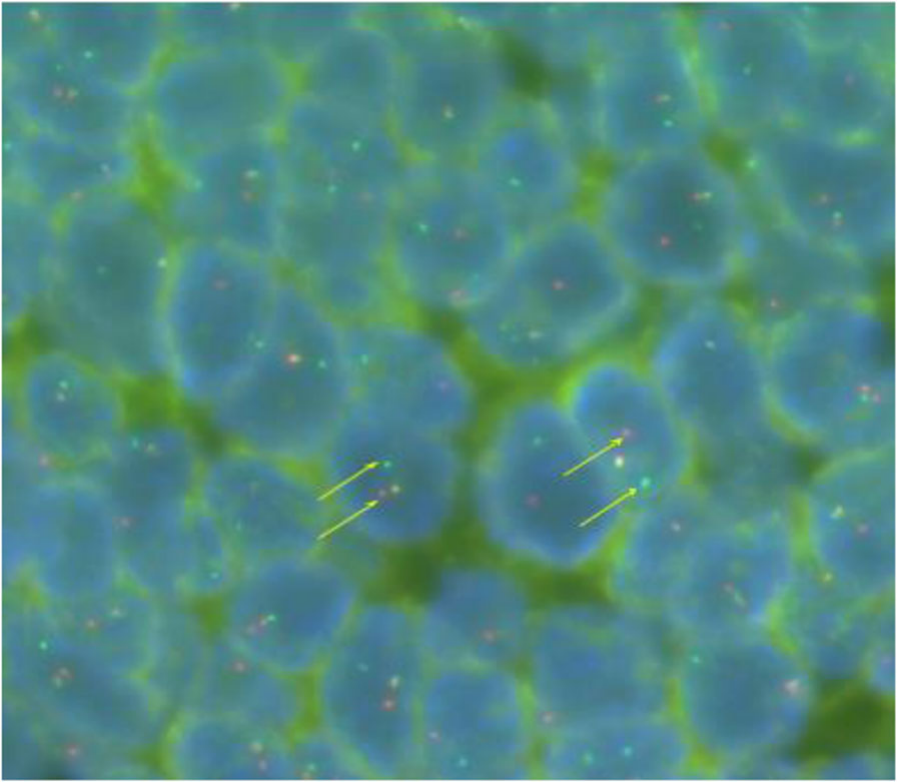
Dual-color, break-apart fluorescence in situ hybridization shows split signals typical of the EWS (22q;12) gene rearrangement. Arrows are pointing to probes (red and green spots) and fusion signal (yellow spots)

The diagnosis of ES was confirmed by molecular study, in situ hybridization revealed the EWSR1 rearrangement gene (Fig. 3).

**Fig. 4:**
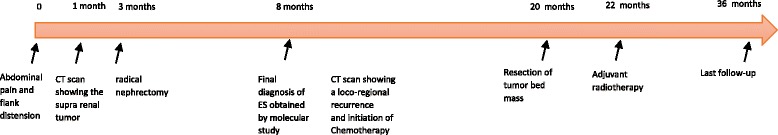
Timeline of patient’s medical history

**Fig. 5:**
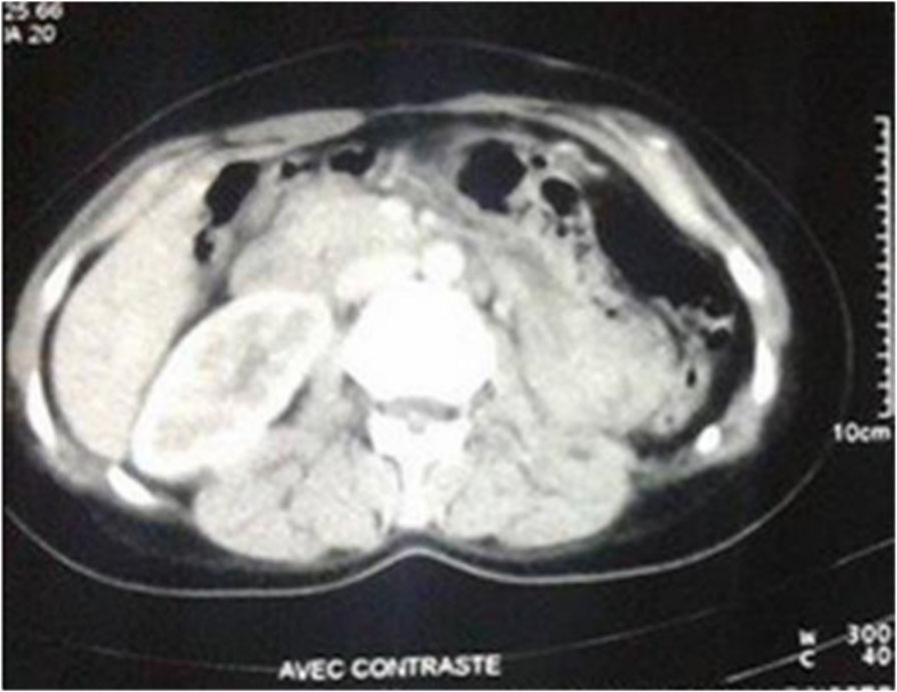
Axial computed tomography performed 5 months after radical nephrectomy showing a heterogeneously enhanced mass of 6 cm in the nephrectomy bed

**Fig. 6:**
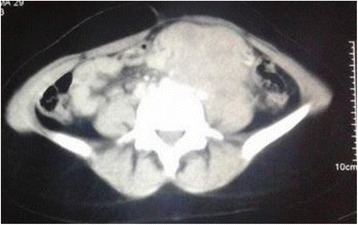
Axial computed tomography performed 5 months after radical nephrectomy showing adenopathies encompassing the left primitive iliac vessels

The final diagnosis was obtained 5 months after surgery due to financial and coordination issues, (Fig. 4), a new imaging in the asymptomatic patient revealed a locoregional recurrence (Figs. 5 and 6).

**Fig. 7:**
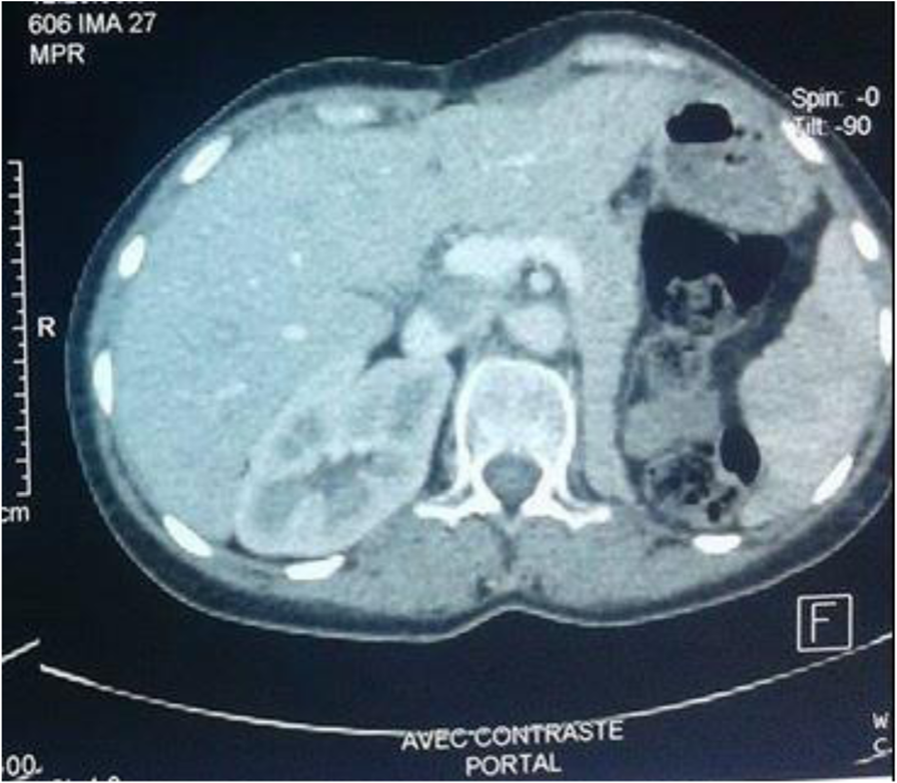
Post-chemotherapy axial computed tomography showing a partial response in the primitive site estimated to be 62%

**Fig. 8:**
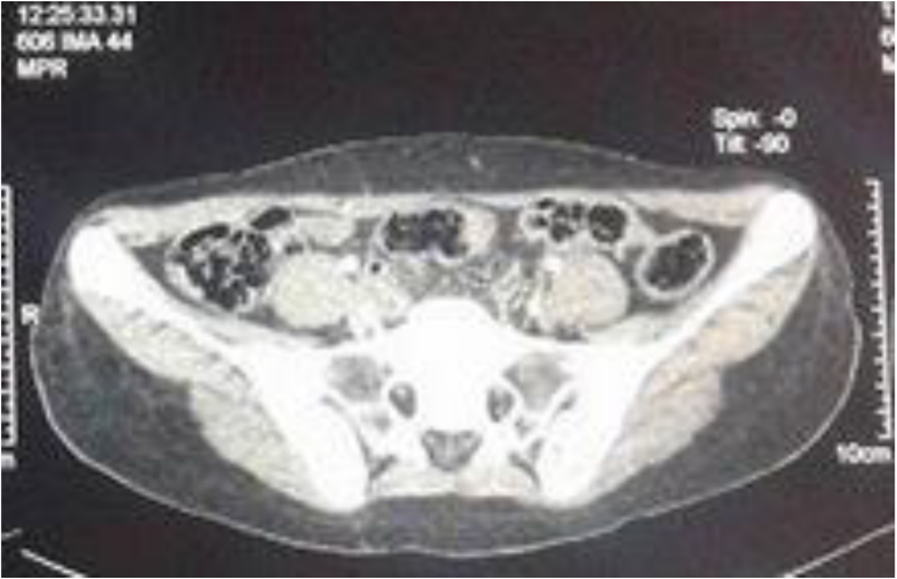
Post-chemotherapy axial computed tomography showing a complete response in lymph nodes

The young woman received 12 cycles of vincristine, doxorubicin and cyclophosphamide (VAC)/ifosfamide and etoposide (IE) alternating every 3 weeks, with partial response estimated to be 62% in the primitive site and a complete response in lymph nodes (Figs. 7 and 8), then underwent a second surgery: resection of recurrent tumor, revealing a totally necrotic 2 cm mass [pathologic complete response (pCR)].

Our patient received adjuvant radiotherapy; 45 Gy was delivered to the tumor bed, para-aortic and left iliac areas, in three-dimensional conformal technique.

After 36 months of follow-up, the young woman is still in remission without any sign of late chemotherapy- or radiotherapy-related toxicity.

## Discussion

In our case report, the patient underwent radical surgery without adjuvant therapy due to financial and interdepartmental coordination issues, diagnosed after 5 months with locoregional recurrence in imaging while she was asymptomatic, than benefited from a neoadjuvant chemotherapy with pathologic complete response, our case report is the only one where recurrence was surgically removed, which gives a precious opportunity to evaluate the pathologic response to chemotherapy. This case confirms the importance of adjuvant treatment and the efficacy of the VAC/IE protocol in this rare localization by extrapolation from osseous Ewing sarcoma management.

Ewing sarcoma of the adrenal gland (ES/PNET) is an extremely rare and aggressive malignancy, only few cases have been reported. It’s worth mentioning that diagnosis of primitive ES in this location is quite difficult, indeed; in case of adrenal mass the metastatic nature must be eliminated, thus an exhaustive imaging should be undergone.

In imaging, ES of adrenal gland presents as a large, well-limited mass, heterogeneously enhanced with areas of hemorrhage and necrosis [1].

The final diagnosis of ES/PNET is not always evident, histologic differential diagnosis of ES could include any small round cell tumor. A typical ES could be diagnosed on the basis of morphologic and immunochemical analysis only; however, cytogenetic confirmation could be needed for final diagnosis. NKX2.2 is considered the best ES marker with a sensitivity of 80% and a specificity of 84%, [2] and so the positivity of CD99 and NKX2.2 is highly specific of ES [2].

Our review of the literature found 28 published cases [3–27], the first case was reported by Marina *et al.* in 1989 [3], our observation is the second case in Morocco to be reported.

Median age at diagnosis was 23.4 years, only three patients were older than 50 years, with a clear female predominance (19 female, 8 male), abdominal pain was the most common revealing symptom. The tumor is usually large, and endocrinologically nonfunctioning. Although there is no standard of care or consensus regarding management of adrenal ES, treatment is extrapolated from osseous ES/PNET management. Current strategy includes local control (surgery and/or radiotherapy) with adjuvant radiotherapy or chemotherapy or both. All patients but two underwent surgery (one patient committed suicide with post-mortem diagnosis, one patient died after neoadjuvant chemotherapy).

Follow-up duration ranged between 6 months and 5 years, four patients had local recurrence with a median progression-free survival (PFS) period of 16 months (1 month in two cases, 9 months in one case and 4.5 years in one case). Five patients had metastatic recurrence involving most often lungs, liver and brain, with a median PFS of 7 months (2 months in one case, 5 months in two cases and 13 months in one case). The patient in the report of Zhang *et al.* [20] with metastatic disease, who did not receive any treatment, died 6 months later.

As shown in the different case reports, adrenal ES/PNET is a rapidly extensive tumor with aggressive behavior. Surgical resection is considered, for the majority of authors, the mainstay of local control. Considering the radio- and chemo-sensitivity of this tumor; adjuvant radiotherapy and chemotherapy are expected to be associated with better disease control by extrapolation to osseous ES, but there is no consensus regarding these adjuvant treatments.

The most common chemotherapy regimen used was VAC/IE for both localized and metastatic disease, other regimens used (for metastatic disease) were: vincristine, actinomycin-D (VAC), adriamycin, cyslophosphamide (AC), platinium, teniposide (PT), prednisolone, cyclophosphamide, vincristine, adriamycin, methotrexate (Pd CVAM), platinium, cyclophosphamide, adriamycin, teniposide (PCAT), vincristine, dacarbazine (VD) and etoposide (E).

None of the studies that has used adjuvant radiotherapy has given details concerning dose and modality but one, the case reported by Yoon *et al*. [11], of a 17-year-old girl who underwent resection with adjuvant sequential chemoradiation. She received 55.8 Gy of proton beam therapy (36 Gy to the abdomen even without capsular rupture, and 19.8 Gy to the tumor bed), with a follow-up of 2 years without any sign of recurrence or sequela.

## Conclusions

Ewing sarcoma of the adrenal gland is an extremely rare and aggressive tumor. Current management includes surgical resection followed by radiotherapy or chemotherapy or both.

A unified therapeutic protocol should be suggested for future cases to point out the best therapeutic sequencing, the appropriate chemotherapy regimen, and radiotherapy volume, dose and timing.

## Abbreviations

A: Adriamycin;
Ac: Actinomycin-D
C: Cyclophosphamide
CT: Computed tomography
D: Dacarbazine
E: Etoposide
ES: Ewing sarcoma
I: Ifosfamide
M: Methotrexate
NSAIDs: nonsteroidal anti-inflammatory drugs
pCR: Pathologic complete response
PFS: Progression-free survival
PNET: Primitive neuroectodermal tumor
PS: Performance status
T: Teniposide
TIRADS: Thyroid Imaging Reporting and Data System
V: Vincristine
